# Novel LARS2 variants in patients with Perrault syndrome: expanding the genetic spectrum and phenotypic heterogeneity

**DOI:** 10.3389/fgene.2026.1785502

**Published:** 2026-02-18

**Authors:** Zibin Lin, Jiale Xiang, Xiangzhong Sun, Xinyu Shi, Xiaozhou Liu, Qinming Cai, Jing Yang, Nana Song, Haodong Ye, Jiangfan Xu, Jiguang Peng, Xianghong Ou, Yu Sun, Zhiyu Peng

**Affiliations:** 1 Reproductive Medicine Center, The Affiliated Guangdong Second Provincial General Hospital of Jinan University, Guangzhou, Guangdong, China; 2 College of Life Sciences, University of Chinese Academy of Sciences, Huairou, Beijing, China; 3 BGI Genomics, Shenzhen, Guangdong, China; 4 Hunan Provincial Key Laboratory of Regional Hereditary Birth Defects Prevention and Control, Changsha Hospital for Maternal and Child Health Care Affiliated to Hunan Normal University, Changsha, Hunan, China; 5 Department of Otorhinolaryngology, Union Hospital of Tongji Medical College, Huazhong University of Science and Technology, Wuhan, Hubei, China

**Keywords:** *LARS2* gene, minigene assay, novel variants, Perrault syndrome, RT-PCR

## Abstract

**Background:**

Perrault syndrome (PS) is a rare autosomal recessive disorder characterized by sensorineural hearing loss (SNHL) and primary ovarian insufficiency in females. *LARS2*, encoding mitochondrial leucyl-tRNA synthetase, is the most common causative gene for PS. However, the genetic spectrum and clinical variability of PS remain underexplored. Expanding the catalog of *LARS2* variants and correlating them with phenotypic data are critical for delineating genotype-phenotype relationships.

**Methods:**

Two unrelated Chinese probands with hearing loss were enrolled, and comprehensive clinical evaluations were performed. Whole-exome sequencing (WES) was used to identify genetic variants, followed by Sanger sequencing for family co-segregation verification. Minigene assays and RT-PCR were conducted to assess the splicing effect of the novel canonical splice-site variant *LARS2* c.235-2A>G. For the novel missense variant *LARS2* c.1661T>C, 3-D structural modeling and evolutionary conservation analysis were performed to evaluate its pathogenicity. Moreover, we comprehensively summarized all *LARS2* variants associated with PS via an extensive literature review.

**Results:**

Proband 1 (12-year-old female) harbors compound heterozygous variants *LARS2* c.235-2A>G (novel) and *LARS2* c.880G>A, presenting with profound SNHL, primary ovarian insufficiency, and developmental delay. Proband 2 (7-year-old male) carries compound heterozygous variants *LARS2* c.1661T>C (novel) and *LARS2* c.1886C>T, manifesting severe SNHL with an unusual upsloping audiogram pattern and comprehension difficulties. Functional assays confirmed that *LARS2* c.235-2A>G disrupts canonical splicing, leading to exon 4 skipping and in-frame deletions. 3-D structural modeling and conservation analysis revealed that *LARS2* c.1661T>C likely impairs protein stability by altering residue interactions, with Val554 being highly conserved across species. According to the ACMG/AMP guideline, both novel LARS2 variants were classified as likely pathogenic.

**Conclusion:**

We identified two novel *LARS2* variants associated with PS in Chinese patients, thereby expanding the *LARS2* genetic spectrum and providing precise molecular evidence for clinical management and genetic counseling. This study enhances understanding of genotype-phenotype correlations in PS, thereby revealing the phenotypic heterogeneity of *LARS2* variants.

## 1 Introduction

Perrault syndrome (PS) is a rare, clinically and genetically heterogeneous disorder manifested as the core features of sensorineural hearing loss (SNHL) in all affected individuals and primary ovarian insufficiency (POI) in females (Carminho-Rodrigues et al., 2020). The syndrome exhibits autosomal recessive inheritance and is genetically complex, with causative mutations identified in several genes, including *HARS2*, *LARS2*, *HSD17B4*, *CLPP*, *ERAL1*, *GGPS1*, *RMND1*, and *TWNK*, each accounting for a subset of cases (Faridi et al., 2022). *LARS2* is the most common causative gene in PS, accounting for 21% of all reported cases (Li et al., 2025).

The *LARS2* gene is located on chromosome 3p21.31. It encodes mitochondrial leucyl-tRNA synthetase. This enzyme is crucial for charging tRNA-Leu with leucine during mitochondrial protein translation. As an essential component of the mitochondrial translation machinery, *LARS2* plays a vital role in oxidative phosphorylation, particularly in high-energy-demanding tissues such as the inner ear and the ovary. Biallelic pathogenic variants in *LARS2* are established as the cause of Perrault syndrome type 4 (OMIM #615300) (Vona, 2023). The clinical features of Perrault syndrome type 4 typically include congenital or early-onset SNHL and POI. Additionally, a significant number of patients also present with diverse neurological manifestations, such as delayed motor development, cognitive impairment, ataxic gait, and tic (Pan et al., 2020; Bayanova et al., 2024). Marfanoid habitus and undervirilization were regarded as nonspecific features of PS (Zerkaoui et al., 2017; Adam et al., 2025).

Notably, only 50 cases with *LARS2* variants have been documented globally, mainly in France, UK, USA, and Australia (Faridi et al., 2022; Lerat et al., 2016; Demain et al., 2017; Riley et al., 2020). Strikingly, only five of these reported cases are from China (Pan et al., 2020; Sun et al., 2022; Lu et al., 2025). This scarcity of reported cases significantly limits our understanding of the genetic spectrum and the associated clinical variability of PS4 in China. Expanding the catalog of *LARS2* variants and correlating them with detailed phenotypic information are therefore crucial to delineate genotype-phenotype correlations and enhance our grasp of the disease’s natural history.

In this study, we identified compound heterozygous *LARS2* variants by whole exome sequencing (WES) in two Chinese patients with hearing loss. Their genotypes included two previously unreported variants: *LARS2* c.235-2A>G and *LARS2* c.1661T>C. In order to precisely evaluate the pathogenicity of these variants, we employed minigene assays and RT-PCR to experimentally validate that the *LARS2* c.235-2A>G variant disrupts normal splicing. For the *LARS2* c.1661T>C variant, we performed 3-D structural modeling and evolutionary conservation analysis, which indicated a deleterious effect on protein stability. Our findings provide robust evidence for the pathogenicity of two novel variants, leading to their classification as “likely pathogenic (LP)”. This work expands the genetic spectrum of *LARS2* and contributes to a refined understanding of genotype-phenotype relationships in this rare syndrome.

## 2 Materials and methods

### 2.1 Patients and clinical investigation

The patients received medical care at the Union Hospital of Tongji Medical College of Huazhong University of Science and Technology. Diagnostic assessments included pure-tone audiometry, blood tests, ultrasonography, magnetic resonance imaging, and so on. This study received approval from the Ethics Committees of Tongji Medical College of Huazhong University of Science and Technology, and BGI.

### 2.2 Exome sequencing and bioinformatic analysis

Genomic DNA was isolated from peripheral blood samples using the Magen DNA extraction kit (Magen Biotech, Guangzhou, China). Qualified genomic DNA samples were processed for library construction. Exome capture was performed using the KAPA HyperExome Probes (Roche, CA, United States), followed by high throughput sequencing at BGI. The sequencing average depth was at least 100x for the target region. Initial processing of the raw sequencing data involved rigorous quality control, followed by the mapping of high-quality reads to the human reference assembly (GRCh37/hg19). To detect single nucleotide variants (SNVs) and insertions/deletions (Indels), we utilized the Genome Analysis Toolkit, while ExomeDepth was integrated into the pipeline specifically for the identification of copy number variations (CNVs) (McKenna et al., 2010; Plagnol et al., 2012).

### 2.3 Variant interpretation and Sanger sequencing validation

The pathogenicity evaluation of SNVs and Indels was conducted in strict accordance with the guideline for the interpretation of sequence variants in genetic hearing loss (Oza et al., 2018). Sanger sequencing was performed to validate the candidate variants identified by WES.

### 2.4 Minigene assay

To validate the molecular impact of *LARS2* c.235-2A>G variant, minigene assay was conducted. A wild-type genomic fragment of *LARS2*, encompassing exon 3 (234 bp) - partial intron 3 (781 bp) - exon 4 (129 bp) - partial intron 4 (448 bp), was amplified from the control’s genomic DNA. The splice variant c.235-2A>G was The wild-type and mutant-type PCR products were cloned into the pcMINI-N vector (Bioeagle Biotech Company, China) at the KpnI and EcoRI restriction sites (Figure 3A). The integrity of recombinant constructs was verified by Sanger Sequencing. HeLa and 293T cells were selected due to their high transfection efficiency and well-characterized splicing profiles. Transient delivery of the recombinant constructs (1 ug per well) was performed using a Rapid Plasmid Mini Kit (1005250, SIMGEN, China) according to the manufacturer’s instructions. Following a 48 h incubation period, total RNA was isolated using the Trizol RNAiso PLUS reagent (9109, TaKaRa, Japan) and subsequently converted into cDNA through reverse transcription. To prevent interference from endogenous *LARS2* transcripts, PCR amplification was carried out using vector-specific flanking primers (pcMINI-N-F: CTA​GAG​AAC​CCA​CTG​CTT​AC and pcMINI-N-R: TAG​AAG​GCA​CAG​TCG​AGG), which specifically target the exogenous transcripts produced by the minigene construct. The amplification products were resolved on agarose gel. Distinct bands corresponding to different splicing isoforms were excised from the gel and subjected to Sanger sequencing to confirm the exact splicing events.

### 2.5 RT-PCR analysis

Peripheral blood samples from family 1 were utilized for total RNA isolation via a commercial extraction kit (TR121-50, Genstone Biotech, China). Subsequently, total RNA was treated with DNase I to remove genomic DNA contamination and reverse-transcribed into cDNA using the Hifair™ first Strand cDNA Synthesis SuperMix (11123ES70, Yeasen, China). To examine the molecular consequences of the *LARS2* c.235-2A>G variant, a pair of primer sets were designed to flank the variant. The sequences of the oligonucleotides used for nested PCR were: *LARS2*-F1: CCT​GTG​AGC​AGA​TCC​AGA​CC; *LARS2*-R1: TTC​CAC​CTT​TGC​TCC​AGA​AC; *LARS2*-F2: ATT​TGA​GGG​CCT​TCT​CAC​CT; *LARS2*-R2: CCC​AGA​CGA​TCA​AGC​TGT​TT. PCR amplification was then carried out using PrimerSTAR MAX DNA Polymerase (R045A, TaKaRa, Japan). The RT-PCR reaction protocol is shown in Supplementary Table S1. The RT-PCR products were verified by Sanger sequencing to assess splicing outcomes.

### 2.6 3-D structural analysis and evolutionary conservation analysis

The UniProt database was utilized to acquire the structure of the wild type LARS2 protein. The tertiary structures of the mutant LARS2 protein were predicted using Missense 3D (https://missense3d.bc.ic.ac.uk/) and then processed and visualized with Pymol. Additionally, evolutionary conservation analysis was performed using Constraint-based Multiple Alignment Tool (https://www.ncbi.nlm.nih.gov/tools/cobalt/cobalt.cgi).

### 2.7 Literature review

A comprehensive literature review was conducted to systematically summarize all reported *LARS2* variants associated with Perrault syndrome. The literature search was conducted using PubMed, Google Scholar, and CNKI databases with keywords including “*LARS2*,” “Perrault syndrome,” and “variant/mutation”. After excluding duplicate reports of the same variant or family, 30 *LARS2* variants were finally included for functional domain mapping and genotype-phenotype correlation analysis.

## 3 Results

### 3.1 Clinical characterization of two probands with Perrault syndrome 4

**FIGURE 1:**
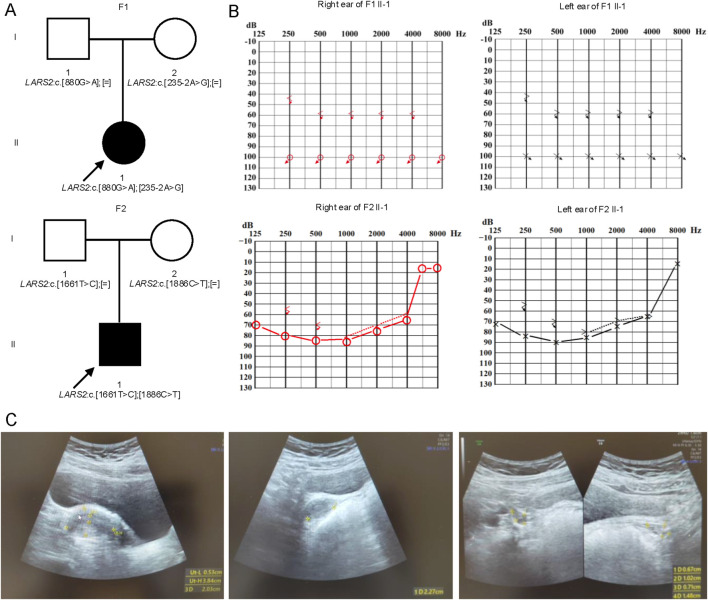
Novel *LARS2* variants in two families affected by hearing loss and/or POI. **(A)** Pedigrees of two families with novel *LARS2* variants. Proband 1 harbors the novel *LARS2* c.235-2A>G *in trans* with *LARS2* c.880G>A. Proband 2 carries the novel *LARS2* c.1661T>C *in trans* with *LARS2* c.1886C>T. **(B)** Audiograms of two probands. Proband 1 exhibits profound hearing loss, and the hearing impairment is similar at both lower frequency and higher frequency. Proband 2 presents with severe hearing loss, and the hearing impairment is more severe at lower frequency. **(C)** Ultrasound features of the ovary and uterus of the proband 1. The patient (F1 II-1) presents with a severe phenotype characterized by uterine hypoplasia and bilateral ovarian hypoplasia with a near-complete absence of antral follicles.

Proband 1 (F1 II-1): She is a 12-year-old daughter of unrelated Chinese parents. She did not pass the newborn hearing screening and was diagnosed with congenital profound SNHL. The patient exhibited delayed development, being able to walk independently at 2 years old. At the age of 5, she underwent right cochlear implantation; and the left ear did not receive a cochlear implant or a hearing aid. Due to the presence of a magnet in her cochlear implant, cranial magnetic resonance imaging is not feasible. Recent pure-tone audiometry revealed bilateral, symmetric, profound hearing loss, with air-conduction thresholds of 100 dB HL and bone-conduction thresholds of 60 dB HL (F1 II-1, Figure 1B). Now she could hear some sounds from her surroundings, but is unable to speak. Now she uses sign language as her primary mode of daily communication. She has poor attention span and limited comprehension ability. Additionally, menarche occurred at 11 years old, with only three menstrual episodes in total, followed by complete amenorrhea to date. Hormonal assays revealed significantly elevated FSH (66.43 IU/L) and LH (29.36 IU/L), along with markedly decreased AMH (0.01 ng/mL) (Table 1). Ultrasonography showed a left ovary measuring 11 mm × 8 mm × 6 mm with no visible follicles, and a right ovary measuring 15 mm × 9 mm × 7 mm with only one follicle larger than 4 mm (Figure 1C). These findings indicate POI. Furthermore, complete blood count, blood glucose, lipid profile, and lactic acid levels were all within normal limits (Table 1).

**TABLE 1:** Clinical characteristics and laboratory profiles of two probands.

Items	F1 II-1	F2 II-1	Reference range
Gender	Female	Male	N/A
Age (years old)	12	7	N/A
Height (cm)	149	122	N/A
Weight (kg)	46.7	22	N/A
Body mass index (kg/m^2^)	21	14.8	N/A
Arm span (cm)	143	121	N/A
Waist circumference (cm)	80	54	N/A
Hemoglobin (g/L)	120	115	113–151
Mean corpuscular volume (fL)	91.5	89.6	77–92
Mean corpuscular hemoglobin (pg)	29.3	29.4	25–34
Mean corpuscular hemoglobin concentration (g/L)	320	328	310–355
Red blood cell distribution width - SD (fL)	45.50	39.60	37–54
Red blood cell distribution width - CV(%)	13.30	12.40	<14.5
Follicle-stimulating hormone (IU/L)	**66.43**	0.75	N/A
Luteinizing hormone (IU/L)	**29.36**	0.01	N/A
Estradiol (pg/mL)	18.00	10.11	N/A
Testosterone (ng/dL)	0.21	<0.13	0.11–0.58
Prolactin (ng/mL)	18.25	10.64	5.18–26.53
Anti-Müllerian hormone (ng/mL)	**0.01**	Not determined	N/A
Glucose (mmol/L)	5.6	4.4	3.9–6.1
Hemoglobin A1c (%)	5.7	5.4	4–6
Cholesterol (mmol/L)	3.54	4.50	<5.2
Triglycerides (mmol/L)	1.74	0.73	<1.7
Lactic acid (mmol/L)	1.6	1.5	0.7–2.1

Data highlighted in bold red and bold blue represent a significant increase and decrease, respectively. Abbreviations: CV, coefficient of variation; N/A, not applicable; SD, standard deviation.

Proband 2 (F2 II-1): He is the 7-year-old son of a non-consanguineous Chinese couple. Newborn hearing screening was not performed. The patient has not received cochlear implantation or hearing aid assistance in either ear. He presented with bilateral, severe SNHL. Recent pure-tone audiometry revealed average air conduction thresholds of approximately 82 dB HL and bone conduction thresholds of 73 dB HL. The audiogram suggested more pronounced hearing loss in 500 Hz and 1,000 Hz (F2 II-1, Figure 1B). Currently, the patient demonstrates poor academic performance and limited comprehension ability. Developmental delay, seizures, or epilepsy were not observed. Cranial MRI showed symmetric brain architecture, preserved gray-white matter differentiation, and no abnormal signal intensities in the parenchyma. Laboratory investigations, including complete blood count, sex hormone profiles, blood glucose, lipid panel, and lactic acid levels, were all within normal limits (Table 1).

### 3.2 Molecular diagnosis: identification of two novel variants in the *LARS2* gene

**FIGURE 2:**
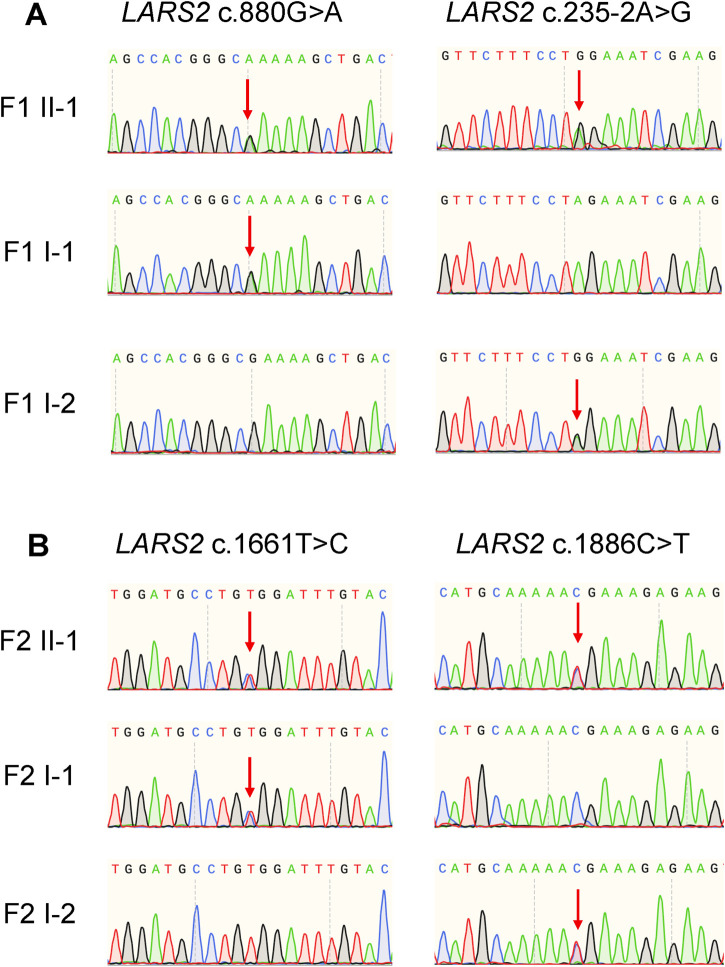
Sanger sequencing validation of *LARS2* variants in two families. **(A)** Sanger sequencing chromatograms of *LARS2* variants in Family 1. The proband (F1 II-1) carries compound heterozygous variants: *LARS2* c.880G>A (inherited from F1 I-1, the father) and *LARS2* c.235-2A>G (inherited from F1 I-2, the mother). **(B)** Sanger sequencing chromatograms of *LARS2* variants in Family 2. The proband (F2 II-1) carries compound heterozygous variants: *LARS2* c.1661T>C (inherited from F2 I-1, the father) and *LARS2* c.1886C>T (inherited from F2 I-2, the mother). Red arrows indicate the variant positions.

Through whole exome sequencing, two variants in the *LARS2* gene were identified in each of the two unrelated probands. Proband 1 carries compound heterozygous variants in the *LARS2* gene: c.880G>A (p.Glu294Lys), which was previously reported by Zazo Seco (Zazo Seco et al., 2016), and a novel variant c.235-2A>G. Proband 2 harbors two additional *LARS2* missense variants: c.1886C>T (p.Thr629Met), which has been documented by Pierce (Pierce et al., 2013), and a second novel variant c.1661T>C (p.Val554Ala). All variants were confirmed by Sanger Sequencing in the respective family members (Figure 2). The parents of each proband are only heterozygous carriers and exhibit normal hearing (Figure 1A). No pathogenic variant was identified in other hearing loss-associated genes.

### 3.3 Aberrant splicing events caused by the novel *LARS2* c.235-2A>G

**FIGURE 3:**
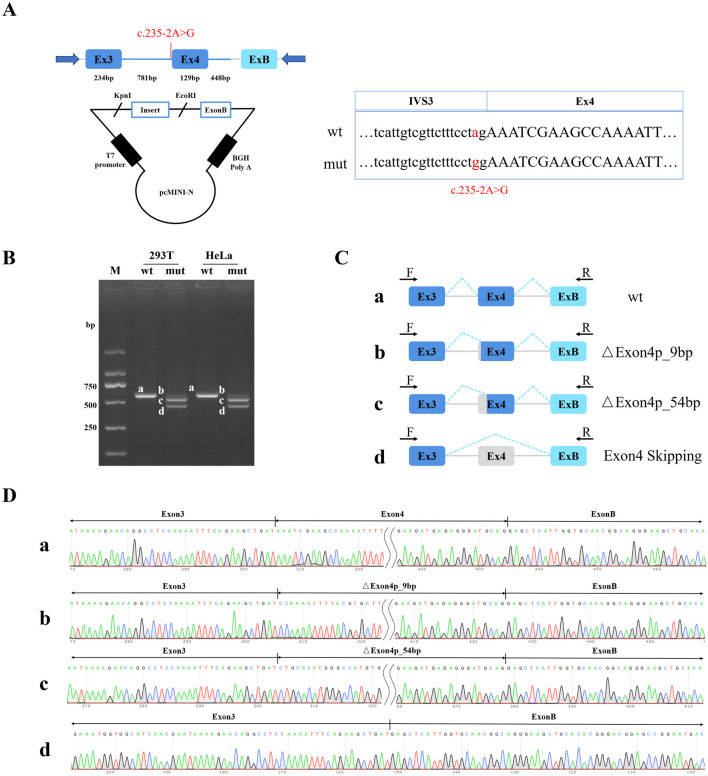
Alternative splicing validation of *LARS2* c.235-2A>G by minigene assay. **(A)** pcMINI-N vector construction strategy map. **(B)** Agarose electrophoresis results of RT-PCR products in 293T and HeLa cells. Bands from wildtype are labeled as a, and bands from c.235-2A>G constructs were labeled as b, c, and d. **(C)** The diagram of alternative splicing events observed in the minigene assay. Band a corresponds to the normal transcript containing exon 4. Band b, c, and d correspond to the transcript with the exon 4 missing the first 9 nucleotides, the exon 4 missing the first 54 nucleotides, and the skipped exon 4, respectively. **(D)** Sanger sequencing results of RT-PCR products.

**FIGURE 4:**
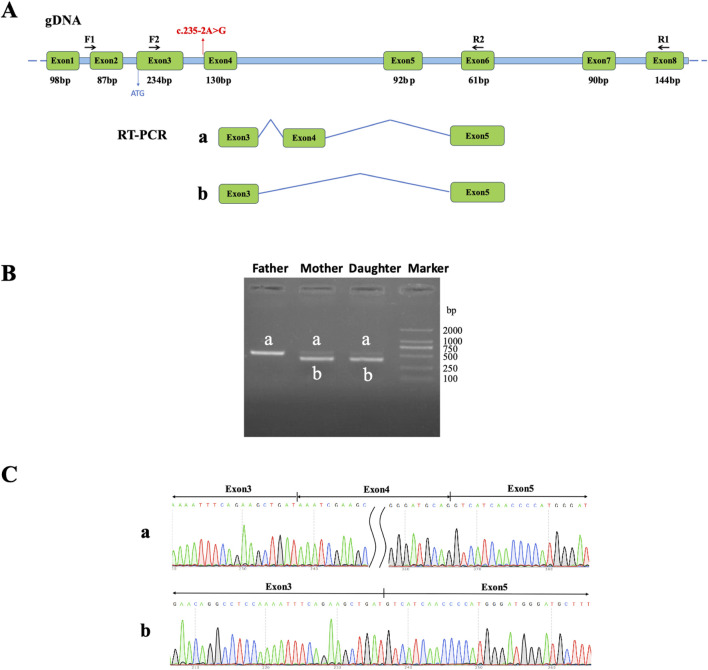
Validation of aberrant splicing of *LARS2* c.235-2A>G from peripheral blood samples. **(A)** Schematic diagram of primer design and splicing events, with red arrows pointing to *LARS2* c.235-2A>G. **(B)** Agarose electrophoresis of RT-PCR products with peripheral leukocytes in family 1. The band amplified from the father was labeled a. Two bands, a and b, were observed in both the mother and daughter. **(C)** Sanger sequencing results of the shear bands. The band a was consistent with the expected size (522 bp), while the band b was abnormal, indicating exon 4 skipping.

Computational analysis via the RDDC^SC^ and Varseak platforms suggested that the *LARS2* c.235-2A>G variant is likely to disrupt a conserved splice acceptor site of exon 4 of *LARS2*, potentially leading to exon skipping. Additionally, SpliceAI indicated that the *LARS2* c.235-2A>G variant leads to the loss of the canonical acceptor site with a high confidence score of 0.99. All three algorithms consistently suggested that this variant is likely to disrupt normal splicing. To functionally assess the impact of this variant on splicing, we performed *in vitro* and *in vivo* experiments. Expression plasmids containing the variant-bearing exon 4 of *LARS2* and its flanking regions were constructed (Figure 3A); these minigene systems were then utilized to assess splicing patterns in 293T and HeLa cellular environments. The variant construct produced aberrant splicing patterns, including a 9-bp deletion within exon 4, a 54-bp deletion spanning part of exon 4, and complete exon 4 skipping (Figures 3B–D). To validate the splicing effect *in vivo*, we performed RT-PCR using peripheral blood from family 1. RT-PCR products from both the proband and her mother who carries the variant only showed exon 4 skipping, whereas her father who does not carry *LARS2* c.235-2A>G exhibits normal inclusion of exon 4 (Figure 4). Both the minigene assay and RT-PCR analysis confirmed that the *LARS2* c.235-2A>G variant led to aberrant splicing.

### 3.4 Interpretation of two novel variants in the *LARS2* gene

This study is the first to report two novel *LARS2* variants (c.235-2A>G and c.1661T>C) associated with PS in the literature. We identified the *LARS2* c.235-2A>G variant in 2023. Although this variant was registered in ClinVar in 2024, it has not been reported in the literature, and no clinical phenotypic information is available for this variant in ClinVar. Additionally, the application of the PVS1 criterion for *LARS2* c.235-2A>G in ClinVar is incorrect.

This study reports a new proband (F1 II-1) who carries *LARS2* c.235-2A>G *in trans* with the likely pathogenic variant *LARS2* c.880G>A, providing additional evidence for the PM3 criterion. Furthermore, the patient presents with POI, which contributes evidence for the PP4 criterion. *LARS2* c.235-2A>G is observed at an extremely low frequency (popmax filtering allele frequency is 0.000019 in East Asian) in the gnomAD dataset, meeting the PM2_Supporting criterion. Notably, *in silico* predictions, minigene assays as well as RT-PCR experiments indicate that *LARS2* c.235-2A>G leads to exon skipping, with the missing protein regions constituting less than 10% of the full-length LARS2 protein. According to the latest PVS1 decision tree, this variant can only be assigned PVS1_Moderate, rather than PVS1 as annotated in ClinVar. Based solely on the information provided in the ClinVar database, the evidence is insufficient to classify the variant as LP. However, when integrated with the proband’s detailed clinical phenotypes and functional experimental validation conducted in this study, the variant meets the criteria for classification as LP (Table 2).

**TABLE 2:** Interpretation of two novel variants in the *LARS2* gene.

Gene	HGVS variant	ACMG/AMP criteria	Classification	Evidence in ClinVar database
*LARS2*	NM_015340.4:c.235-2A>G	PM2_Supporting, PVS1_Moderate, PM3, PP4	LP	PVS1, PM2_Supporing
*LARS2*	NM_015340.4:c.1661T>C	PM2_Supporting, PP3, PM3_Strong, PP4	LP	Not listed

*LARS2* c.1661T>C does not exist in gnomAD, meeting the PM2_Supporting criterion. Computational prediction tools support a deleterious effect of the *LARS2* c.1661T>C variant on the gene, providing the PP3 criterion. Moreover, this study reports two unrelated hearing loss patients who carry *LARS2* c.1661T>C *in trans* with *LARS2* c.1886C>T (another pedigree is shown in Supplementary Figure S1), providing PM3_Strong evidence. In addition to SNHL, the patient exhibits academic challenges and impaired comprehension, which aligns with the PP4 evidence criterion. According to the ACMG/AMP guideline, the *LARS2* c.1661T>C variant was classified as LP (Table 2).

### 3.5 3-D structural analysis and conservation analysis of the novel *LARS2* c.1661T>C

**FIGURE 5:**
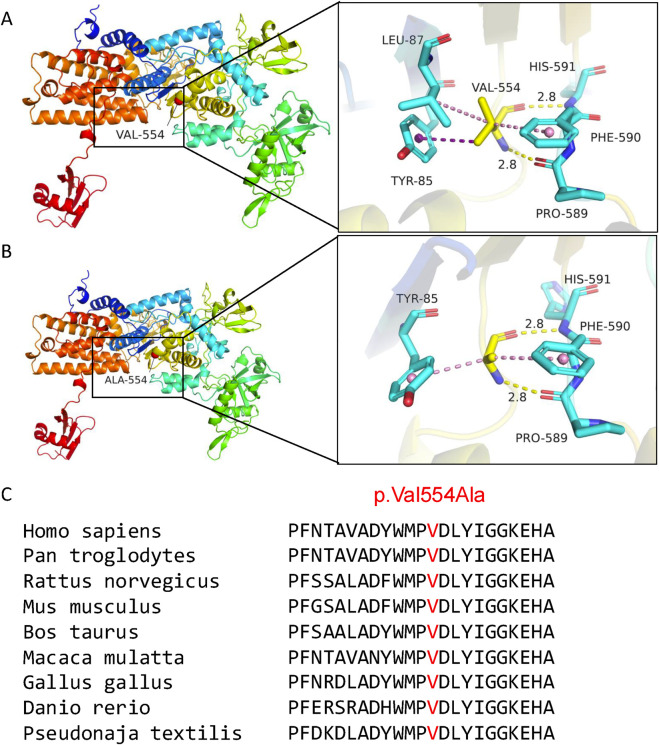
Structural analysis and conservation analysis of the novel *LARS2* c.1661T>C (p.Val554Ala). In the wild-type **(A)**, the side chain of Val554 residue engages in Pi-Alkyl/Alkyl interactions with Phe590 and Leu87, and its methyl group establishes a Pi-sigma interaction with the benzene ring of Tyr85. In the mutant **(B),** the methyl group of side chain forms Pi-Alkyl interactions with Phe590 and Tyr85. **(C)** The missense variant *LARS2* p.Val554Ala occurs at a highly conserved residue across nine species.

Three-dimensional structural modeling was performed to assess the pathogenic potential of *LARS2* c.1661T>C (p.Val554Ala). The p.Val554Ala variant is located within the catalytic domain of *LARS2*. This substitution replaces valine with alanine, converting the side chain from an isopropyl group to a smaller methyl group. Although both the wild-type (valine) and mutant (alanine) residues are non-polar and form backbone hydrogen bonds with HIS591 and PRO589 at distances of 2.8 Å each, the Val-to-Ala change is predicted to alter side chain interactions with surrounding residues (Figures 5A,B). This disruption is likely to decrease the protein stability, and potentially impair aminoacylation efficiency.

Evolutionary conservation analysis reveals that the valine residue at position 554 is highly conserved across diverse species, including mammals and non-mammals (Figure 5C). This finding suggests a critical role of Val554 in the LARS2 protein’s structure and function.

### 3.6 The variant spectrum and domain architecture of *LARS2* variants

**FIGURE 6:**
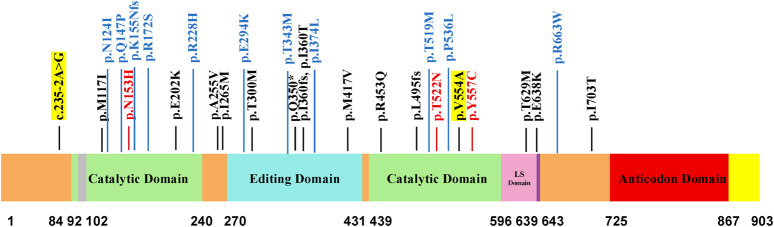
Domain distribution of *LARS2* variants and their genotype-phenotype correlations. Color-coded variants represent distinct clinical manifestations. Black variants are associated with hearing loss and/or POI; blue variants present these phenotypes plus neurological symptoms; red variants present with additional Marfanoid features. Novel variants identified in this study are highlighted with yellow backgrounds. The schematic diagram displays functional domains of *LARS2* protein with amino acid positions indicated below.

Through a comprehensive literature review, we systematically mapped the distribution of 30 *LARS2* variants onto the functional domains of LARS2 protein (Figure 6). 15 variants were predominantly localized to the catalytic domain, which is responsible for amino acid activation and tRNA aminoacylation. Eight variants were localized to the editing domain, which ensures translational fidelity by proofreading tRNAs.

Moreover, genotype-phenotype analysis revealed that all individuals harboring P/LP *LARS2* variants presented with deafness and/or POI, with a substantial subset exhibiting additional neurological symptoms. Notably, specific variants (p.N153H, p.T522N, p.Y557C) were associated with Marfanoid features. The novel *LARS2* c.235-2A>G, a splice-site variant in intron 3 preceding the catalytic domain, was identified in a 12-year-old girl presenting with both profound SNHL and POI. The novel *LARS2* p.Val554Ala, located within the catalytic domain, was found in a 7-year-old boy with hearing loss accompanied by learning and comprehension difficulties.

## 4 Discussion

We successfully identified two novel *LARS2* variants—c.235-2A>G and c.1661T>C (p.Val554Ala)—in two unrelated Chinese probands with PS. Functional assays confirmed that the c.235-2A>G variant disrupts normal splicing, and structural modeling indicated that the p.Val554Ala substitution likely impairs protein stability. In accordance with the ACMG/AMP guideline (Richards et al., 2015), both variants were classified as LP. Our findings contribute to the genetic heterogeneity of *LARS2*-related Perrault syndrome and provide valuable insights into the genotype-phenotype correlations of this rare disorder.

Based on comprehensive genetic, phenotypic, and biochemical analyses, two probands in this study were diagnosed with Perrault syndrome 4 (Al-Jaroudi et al., 2019; Lerat et al., 2016) instead of hydrops, lactic acidosis, and sideroblastic anemia (OMIM #617021). Both probands were diagnosed with SNHL and POI, and they had normal lactic acid levels and unremarkable hematological profiles (Table 1). Consistent with previous reports (van der Knaap et al., 2019; Neyroud et al., 2023), proband 1 in our study mirrored the core phenotypes in affected females—congenital SNHL, delayed motor milestones, and POI. Proband 1 carries compound heterozygous *LARS2* variants (c.880G>A and c.235-2A>G) and exhibits congenital profound SNHL. However, Pan et al. reported a Chinese patient with progressive hearing loss harboring compound heterozygous variants of *LARS2* (c.880G>A and c.2108T>C). This patient was diagnosed with bilateral moderate SNHL at 2 years of age, which progressed to severe hearing loss by 22 years old (Pan et al., 2020). Notably, both patients share the c.880G>A variant, so the difference in hearing loss severity and onset between the two patients may be attributed to their respective second variants: the canonical splice variant c.235-2A>G induces deletion of exon 4, while the missense variant c.2108T>C only results in a single amino acid change. Importantly, exon 4 is part of the catalytic domain of LARS2 protein–a domain critical for mitochondrial tRNA binding. Thus, partial deletion of this domain may significantly disrupt mitochondrial function in auditory tissues, thereby contributing to the more severe and earlier-onset hearing phenotype.

Notably, proband 1 received cochlear implantation of her right ear at age 5, but showed no language development at age 12. This may be due to *LARS2* variants potentially causing leukodystrophy. Although there is currently no cranial magnetic resonance imaging data to directly confirm the presence of leukodystrophy, proband 1 exhibits developmental delay and impaired comprehension—phenotypes that align closely with previous reports linking *LARS2* variants to leukodystrophy. Such leukodystrophy may affect auditory signal transmission and processing (van der Knaap et al., 2019). As a result, proband 1 only achieved environmental sound perception, with limited speech recognition ability and less effectiveness of cochlear implantation. This finding carries important clinical implications for patients with SNHL caused by *LARS2* variants. Cochlear implantation may not yield significant therapeutic benefits in terms of language development, suggesting that clinicians should exercise caution when recommending cochlear implantation for such patients. Meanwhile, this case also highlights the crucial role of genetic testing in the clinical management of patients with congenital SNHL. Furthermore, given the genetic etiology of POI, her parents consulted about ovarian cortex cryopreservation to preserve her fertility, which holds significant importance for safeguarding her reproductive potential.

Proband 2 presents with hearing loss accompanied by academic challenges and limited comprehension ability. In contrast, Sun et al. reported two Chinese patients with compound heterozygous *LARS2* variants (c.764C>T and c.1987C>T) who exhibited isolated hearing loss (Sun et al., 2022). The phenotypic discrepancy may be attributed to the distinct positions of the variants: neither c.764C>T nor c.1987C>T resides within the key functional domains of the LARS2 protein, whereas c.1661T>C and c.1886C>T are situated in the catalytic domain and LS domain—core regions critical for LARS2 protein function. Consequently, in addition to hearing loss, proband 2 displays neurodevelopmental involvement at 7 years old. Previous studies reported that more severe neurological manifestations associated with pathogenic *LARS2* variants, such as seizures, progressive cognitive impairment, and leukodystrophy, mostly occur in patients around 30–40 years of age (van der Knaap et al., 2019). The phenotypic discrepancy between proband 2 and previously reported cases suggests that the academic challenges and limited comprehension ability in proband 2 may represent an early-onset and milder form of *LARS2*-associated neurological involvement. This not only expands the phenotypic spectrum of *LARS2*-related PS, but also reveals the phenotypic heterogeneity of *LARS2* variants in different age groups. Consequently, regular neurological monitoring and tailored rehabilitation are recommended to mitigate potential functional decline. Notably, the audiogram of proband 2 indicated more pronounced hearing loss at lower frequencies (500–1,000 Hz), resulting in an unusual upsloping audiogram, a feature previously associated with the *LARS2* c.1565C>A variant (Adam et al., 2025; Demain et al., 2017). The novel *LARS2* c.1661T>C variant lies in close proximity to *LARS2* c.1565C>A, and both variants are associated with an unusual upsloping audiogram, suggesting that this specific protein region may be critical for determining this particular audiological phenotype.

At the molecular level, we conducted functional validation for the novel variants. For the splice-site variant c.235-2A>G, the minigene assay in 293T and HeLa cells revealed three aberrant splicing events (a 9-bp deletion, a 54-bp deletion within exon 4, and complete exon 4 skipping), whereas RT-PCR from the patient’s peripheral blood predominantly showed complete exon 4 skipping. This discrepancy is likely attributed to cell-type-specific differences in the expression of splice factors and splice regulatory factors (van der Klift et al., 2015). 293T and HeLa cells are immortalized lines that may possess a different landscape of splicing factors (such as SR proteins and hnRNPs) compared to primary peripheral blood cells, which can lead to the recognition of cryptic splice sites in a heterologous expression system (Holmes and Hertel, 2026; Waks et al., 2011; Zhang et al., 2025). Critically, all aberrant transcripts are predicted to cause partial or complete deletion of exon 4, with the missing protein regions constituting less than 10% of the full-length LARS2 protein. According to the ACMG/AMP guideline, all the observed splicing effects consistently meet the criteria for PVS1_Moderate (Abou Tayoun et al., 2018). Consequently, both the minigene assay and patient derived RT-PCR confirmed consistent pathogenicity ratings for the c.235-2A>G variant, supporting its classification as LP.

Collectively, the patients’ clinical phenotypic findings and functional experimental data enabled definitive molecular diagnosis of the two cases. These findings bear significant implications for clinical practice and genetic counseling. First, our results reinforce the clinical imperative of including *LARS2* in genetic testing panels for hearing loss. Second, rigorous pathogenicity assessment of these novel variants has afforded the affected families an accurate molecular diagnosis, thereby facilitating genetic counseling and risk stratification for at-risk family members. In Family 1, the proband’s sister was confirmed to be a heterozygous carrier with normal hearing and ovarian function, which has markedly alleviated familial anxiety regarding disease recurrence. Furthermore, the parents of both probands, upon receiving a clear genetic diagnosis, are planning to utilize preimplantation genetic testing for their next pregnancy. Our results directly underpin this critical reproductive decision-making process.

We acknowledge that the primary limitation of this study is its small sample size, involving only two families. This inherent constraint may limit our ability to provide a truly comprehensive description of the expansive and heterogeneous phenotypic spectrum associated with *LARS2* variants. To address this constraint, we emphasize that larger multi-center cohort studies (encompassing both Chinese and international populations) are essential for future research. Such expanded cohorts will help us understand the genotype-phenotype correlations, clarify the clinical heterogeneity of *LARS2* variants, and provide more robust evidence to guide clinical management of affected individuals. Furthermore, elucidating the molecular mechanism by which *LARS2* c.1661T>C predisposes to unusual upsloping audiogram could provide deeper insights into genotype-phenotype correlations.

In conclusion, our study identifies and functionally characterizes two novel *LARS2* variants, expanding the genetic architecture of PS. We demonstrate that the c.235-2A>G variant causes aberrant splicing and that the c.1661T>C variant is likely to decrease protein stability. This work underscores the necessity of comprehensive analysis for the accurate interpretation of variants of uncertain significance and enhances our understanding of the clinical and genetic landscape of *LARS2*-related disorders. This study elucidates the molecular etiology of the patients, facilitating precise next-step clinical management and informing future reproductive decisions.

## Data Availability

All data supporting the findings of this study are available either within the article, Supplementary Material, or from the corresponding authors upon reasonable request. The data are not publicly available due to privacy or ethical restrictions.
