# Supplementary material for: Novel LARS2 variants in patients with Perrault syndrome: expanding the genetic spectrum and phenotypic heterogeneity
Source: Front Genet. 2026 Feb 18;17:1785502. doi: 10.3389/fgene.2026.1785502 (PMC12956261; doi:10.3389/fgene.2026.1785502)
Supplement: Supplementary file 1 [file DataSheet1.docx]

**Supplementary Table 1** RT-PCR reaction protocol

| **Step** | **Temperature** | **Time** | **Cycles** |
| --- | --- | --- | --- |
| 1 | 95 °C | 5 min | 1 |
| 2 | 95 °C | 30 s | 35 |
| 3 | 57 °C | 30 s |  |
| 4 | 72 °C | 90 s |  |
| 5 | 72 °C | 5 min | 1 |

**Supplementary Figure 1** Another pedigree carries the novel *LARS2* c.1661T>C variant. The proband is 5 years old and presents with congenital, bilateral sensorineural hearing loss. His parents refused further follow-up after completing genetic testing.
